# Association between single nucleotide polymorphisms in ADRB2, GNB3 and GSTP1 genes and high-altitude pulmonary edema (HAPE) in the Chinese Han population

**DOI:** 10.18632/oncotarget.15309

**Published:** 2017-02-14

**Authors:** Yongjun He, Lijun Liu, Pengcheng Xu, Na He, Dongya Yuan, Longli Kang, Tianbo Jin

**Affiliations:** ^1^ Key Laboratory of High Altitude Environment and Genes Related to Diseases of Tibet Autonomous Region, School of Medicine, Xizang Minzu University, Xianyang, Shaanxi 712082, China; ^2^ Key Laboratory for Molecular Genetic Mechanisms and Intervention Research on High Altitude Disease of Tibet Autonomous Region, School of Medicine, Xizang Minzu University, Xianyang, Shaanxi 712082, China; ^3^ Key Laboratory for Basic Life Science Research of Tibet Autonomous Region, School of Medicine, Xizang Minzu University, Xianyang, Shaanxi 712082, China; ^4^ Department of Hand Surgery, Hebei Province Cangzhou Hospital of Integrated Traditional and Western Medicine, Cangzhou, Hebei 061001, China

**Keywords:** GNB3, GSTP1, ADRB2, high-altitude pulmonary edema, genetic

## Abstract

High altitude pulmonary edema (HAPE) occurs mainly under conditions such as high altitude, rapid ascent, or hypoxia. Previous studies suggest that *ADRB2*, *GNB3*, *TH*, and *GSTP1* polymorphisms are associated with various lung diseases. We evaluated whether those polymorphisms are associated with the risk of HAPE in a Chinese Han population. *ADRB2*, *GNB3*, *TH* and *GSTP1* polymorphisms were genotyped using a Sequenom MassARRAY. Logistic regression, adjusted for age and gender, was used to evaluate the association between the genotypes and the risk of HAPE by computing odds ratios (ORs) and 95% confidence intervals (95% CIs). The results revealed that *GNB3* rs4963516 allele ‘‘G’’ (G vs T: OR = 0.70, 95% CI = 0.55–0.90, *p* = 0.006) was associated with HAPE risk. The *ADRB2* rs1042718 alleles had a 1.29-fold (95%CI = 1.00-1.66; *p* = 0.045) increased risk of HAPE, and the *GSTP1* rs749174 alleles had a 0.71-fold (95%CI = 0.52-0.99; *p* = 0.042) decreased risk of HAPE. Co-dominant and dominant models of *GNB3* rs4963516 decreased the risk of HAPE (*p* = 0.023 and *p* = 0.008, respectively). Our results indicate *GNB3* and *GSTP1* polymorphisms may protect against HAPE progression, while *ADRB2* polymorphisms are associated with an increased risk of HAPE.

## INTRODUCTION

High-altitude pulmonary edema (HAPE), a potentially fatal medical condition, is the cause of deaths due to high-altitude illness [1]. HAPE is a form of increased permeability pulmonary edema of non-cardiogenic origin that usually occurs within 2-4 days of ascent above 2,500-3,000 m [1, 2]. Patients with HAPE include two types: inhabitants of high-altitudes who return to low altitudes, also known as “re-entry” HAPE, and healthy lowlanders who rapidly climb up and are not acclimatized to high altitudes [3]. In HAPE, hypoxia-induced pulmonary vasoconstriction leads to endothelial dysfunction and intravascular fluid retention [4, 5]. HAPE occurs frequently in people exposed to high altitudes [6, 7]. More than 140 million people living in high-mountain areas, including Asia and Americas, have suffered from HAPE [3]. Recently, the HAPE mortality rates have reached 50% [8], because of the increase in skiing, trekking, and climbing tours.

HAPE is a multifactorial disease that involves both environmental and genetic factors. Studies indicate that cell and tissue hypoxia can promote gene expression and induce expression of physiologically important proteins [9–13]. The genetic sensitivity to hypoxia has been known for years, and includes genes, such as *nitric oxide synthase* (*NOS*) [9], *tyrosine hydroxylase* (*TH*) [14], *angiotensin-converting enzyme* (*ACE*) [10], *endothelin-1* (*EDN-1*) [11, 12], and *vascular endothelial growth factor* (*VEGF*) [13]. Genetic single nucleotide polymorphisms (SNPs) play a pivotal role in high altitude diseases, including the HAPE risk [15]. However, genetic studies about the etiology of HAPE are still rare.

In our study, we selected 15 SNPs in *beta-2 adrenergic receptor (ADRB2), G protein β3 subunit (GNB3)*, *glutathione transferase Pi 1* (*GSTP1*), and *TH* genes, which have been reported to be associated with pulmonary diseases, including asthma, HAPE, lung cancer, and chronic obstructive pulmonary disease (COPD) [16–20]. Based on previous research, we conducted a case-control study and identified an association of the *ADRB2*, *GNB3*, *TH*, and *GSTP1* genes with the risk of HAPE in Chinese Han population. These results may provide a basis for the clinical prevention of HAPE.

## RESULTS

A total of 267 patients with HAPE and 304 healthy people were enrolled in this case-control study. The HAPE cases and controls were matched by sex, but there was a significant difference in age between HAPE cases and controls (*p* < 0.05); the average ages were 36.2 ± 4.5 years and 32.6 ± 10.7 years, respectively. The characteristics of the studied population are summarized in Table 1.

**Table 1:** Characteristics of the subjects

variable	case	control	*p*
Number	267	304	
sex			> 0.001
male	246	290	
female	21	14	
age, year (mean ± SD)	32.57±10.74	36.15±4.45	< 0.001*

* *p* < 0.05 indicates statistical significance

Table 2 summarizes the basic information of candidate SNPs in our study, such as chromosomal position, gene, allele, Hardy-Weinberg equilibrium (HWE) test results, and minor allele frequency (MAF). The HWE was agreed by *p* > 0.05. All success rates of the genotype assays were designed to have the cut off level less 98%.

**Table 2:** Allele frequencies in cases and controls and odds ratio estimates for HAPE risk

SNP	Gene	Band	Base Change	MAF-case	MAF-control	*p* value for HWEtest	OR	95%CI		*p* value
rs17778257	*ADRB2*	5q32	A/T	0.36	0.35	0.38	1.03	0.81	1.31	0.827
rs2400707	*ADRB2*	5q32	A/G	0.29	0.34	0.80	0.81	0.63	1.04	0.093
rs17108803	*ADRB2*	5q32	G/T	0.05	0.05	0.48	0.98	0.56	1.71	0.940
rs12654778	*ADRB2*	5q32	A/G	0.36	0.34	0.31	1.06	0.83	1.35	0.642
rs11168070	*ADRB2*	5q32	C/G	0.09	0.09	0.73	0.95	0.63	1.43	0.815
rs1042718	*ADRB2*	5q32	A/C	0.36	0.30	0.68	1.29	1.01	1.67	0.045*
rs3842727	*TH*	11p15.5	G/T	0.04	0.04	1.00	0.95	0.52	1.73	0.860
rs2070762	*TH*	11p15.5	A/G	0.39	0.36	0.14	1.13	0.89	1.44	0.311
rs10770140	*TH*	11p15.5	C/T	0.09	0.09	1.00	1.10	0.74	1.66	0.632
rs10770141	*TH*	11p15.5	A/G	0.08	0.08	0.24	1.00	0.66	1.53	0.992
rs10840491	*TH*	11p15.5	A/G	0.10	0.09	1.00	1.11	0.74	1.66	0.611
rs7119275	*TH*	11p15.5	C/T	0.09	0.09	0.15	1.06	0.70	1.59	0.795
rs749174	*GSTP1*	11q13.2	A/G	0.14	0.18	0.85	0.72	0.52	0.99	0.042*
rs4963516	*GNB3*	12p13.31	G/T	0.29	0.37	0.27	0.70	0.55	0.90	0.006*
rs5446	*GNB3*	12p13.31	C/T	0.20	0.20	0.15	1.02	0.76	1.36	0.914

MAF= minor allele frequency; HWE= Hardy–Weinberg Equilibrium; OR =odds ratio; 95 % CI = 95 % confidence interval;

*p < 0.05 indicates statistical significance

We compared the frequency of alleles distributions and found that three SNPs were associated with HAPE risk in three genes (rs1042718 in *ADRB2*; OR = 1.29, 95 %CI = 1.00–1.67, *p* = 0.044; rs749174 in *GSTP1*; OR = 0.71, 95 %CI = 0.52–0.99, *p* = 0.041; and rs4963516 in *GNB3*; OR = 0.70, 95 %CI = 0.55–0.90, *p* = 0.006).

The results of the genetic model are shown in Table 3. Co-dominant, dominant, recessive, and log-additive models were calculated and adjusted by age and gender. We found that co-dominant (*p* = 0.023) and dominant models (GT/GG vs TT: OR = 0.63, 95%CI = 0.45 – 0.89, *p* = 0.008) in *GNB3* rs4963516 were associated with the risk of HAPE. We did not find any statistically significant association between the genotype and genetic model in other SNPs and the risk of HAPE. No association was observed after Bonferroni correction.

**Table 3:** Single-SNP analysis (rs1042718, rs749174 and rs4963516) under different genetic models (adjusted by age and gender)

SNP	Model	Genotype	control	case	OR (95% CI)	*p*
rs1042718	Co-dominant	C/C	144 (49%)	110 (43.5%)	1	0.140
		C/A	121 (41.2%)	103 (40.7%)	1.12 (0.77-1.62)	
		A/A	29 (9.9%)	40 (15.8%)	1.76 (1.01-3.06)	
	Dominant	C/C	144 (49%)	110 (43.5%)	1	0.230
		C/A-A/A	150 (51%)	143 (56.5%)	1.24 (0.88-1.76)	
	Recessive	C/C-C/A	265 (90.1%)	213 (84.2%)	1	0.056
		A/A	29 (9.9%)	40 (15.8%)	1.67 (0.98-2.82)	
	Log-additive	---	---	---	1.26 (0.98-1.62)	0.072
rs4963516	Co-dominant	T/T	126 (41.7%)	138 (51.7%)	1	0.023*
		G/T	131 (43.4%)	104 (39%)	0.66 (0.46-0.95)	
		G/G	45 (14.9%)	25 (9.4%)	0.54 (0.31-0.93)	
	Dominant	T/T	126 (41.7%)	138 (51.7%)	1	0.008*
		G/T-G/G	176 (58.3%)	129 (48.3%)	0.63 (0.45-0.89)	
	Recessive	T/T-G/T	257 (85.1%)	242 (90.6%)	1	0.110
		G/G	45 (14.9%)	25 (9.4%)	0.65 (0.38-1.10)	
	Log-additive	---	---	---	0.71 (0.55-0.91)	0.110
rs749174	Co-dominant	G/G	201 (66.8%)	198 (74.2%)	1	0.190
		A/G	91 (30.2%)	65 (24.3%)	0.74 (0.51-1.09)	
		A/A	9 (3%)	4 (1.5%)	0.50 (0.15-1.66)	
	Dominant	G/G	201 (66.8%)	198 (74.2%)	1	0.086
		A/G-A/A	100 (33.2%)	69 (25.8%)	0.72 (0.50-1.05)	
	Recessive	G/G-A/G	292 (97%)	263 (98.5%)	1	0.300
		A/A	9 (3%)	4 (1.5%)	0.54 (0.16-1.79)	
	Log-additive	---	---	---	0.73 (0.52-1.03)	0.067

OR =odds ratio; 95 %CI = 95 % confidence interval;

*p < 0.05 indicates statistical significance

Finally, in order to assess the association between SNP haplotypes and HAPE risk, a Wald test was performed using an unconditional multivariate regression analysis. However, no association was observed (data not shown).

## DISCUSSION

In this study, we hypothesized that the *ADRB2, GNB3, TH*, and *GSTP1* polymorphisms are associated with the HAPE risk in Chinese Han population. Our results show that the alleles “G” in *GNB3* rs4963516 (*p* = 0.006), “A” in *ADRB2* rs1042718 (*p* = 0.045), and “A” in *GSTP1* rs749174 (*p* = 0.041) are associated with the risk of HAPE.

The *GNB3* gene, located in 12p13, has been associated with mountain sickness [17]. It has been also reported in the hypertensive crisis, cardiovascular disease, and digestive system cancer [21, 22]. *GNB3* regulates heterotrimeric guanine nucleotide-binding proteins (G-proteins), which integrate signals between receptors and effector proteins. HAPE is characterized by increased water and salt metabolism, and by abnormal cell membrane ion transport, which are regulated by G-proteins and beta 2 adrenergic receptor protein. In this study, we found that co-dominant and dominant models of *GNB3* rs4963516 are associated with the decreased risk of HAPE. In contrast, we did not find a statistically significant correlation between rs5446 in *GNB3* gene and the risk of HAPE; this is consistent with a previous study [23].

We used Hap Map to identify SNPs in the *ADRB2* gene, a small (3446bp transcript, 1239bp coding sequence), intronless gene on chromosome 5 (Gene-Card number GC05P148186), which is the principal catecholamine receptor in the lungs, and plays a crucial role in pulmonary diseases. Wang *et al* reported that the common haplotypes in the *ADRB2* are not associated with acute mountain sickness susceptibility in Nepalese population [24], while Chandramoorthi *et al* showed a significant association between the *ADRB2* gene and the HAPE risk [25]. In this study, we found that the allelic model in *ADRB2* rs1042718 is associated with the risk of HAPE (Table 2). However, our results show that the genetic models of *ADRB2* are not associated with the risk of HAPE.

*GSTP1* gene has been associated with various cancers, including breast, lung, prostate, bladder, and esophageal cancer [26]. In addition, Mishra *et al* have shown a correlation between the *GSTP1* gene and the risk of HAPE [27]. Our study indicates that the polymorphism of *GSTP1* rs749174 is associated with the risk of HAPE.

Consistent with previous studies, we have found no significant correlation between the blunted hypervariable region values in HAPE with the polymorphisms of the *TH* gene. These results indicate that the pathogenesis of HAPE is not influenced by the mutations of the *TH*, and that these polymorphisms may not be suitable genetic markers for the HAPE-susceptibility.

A limitation of this study is the relatively small data set, which limits the statistical power to detect effects for several of the assessed SNPs, particularly since much of them are not frequent. In addition, while the potential risk factors for HAPE, such as body mass index, menopausal status, smoking, and age, may interact with the SNPs, the relative similarity of our patient and control populations has reduced any confounding effects. Using Bonferroni correction, no statistically significant association between SNPs and the risk of HAPE has been found. This may be due to the relatively small sample size, the selection criteria for SNPs (minor allele frequency > 5%), and the weakness of the Bonferroni correction itself (the result interpretation depends on the number of other tests performed). True differences may have been deemed non-significant given the likelihood of type II errors.

To further evaluate the effect of polymorphisms of *ADRB2, GNB3* and *GSTP1* genes on the HAPE risk, future studies should analyze gene-gene and gene-environment interactions using large sample sizes.

## CONCLUSION

This study demonstrates the association between the risk of HAPE and the polymorphism of *ADRB2*, *GNB3*, *TH*, and *GSTP1* genes. Polymorphisms of *GNB3* and *GSTP1* genes may be a potential protective factor in the HAPE progression in Chinese Han population. In contrast, *ADRB2* may increase the risk of HAPE. Additional studies are needed to confirm this association, and clarify the role of *ADRB2, GNB3* and *GSTP1* genes in the HAPE pathogenesis.

## MATERIALS AND METHODS

### Study participants

Of the 571 participants, 267 were diagnosed with HAPE and recruited between August 2013 and December 2015 at the Hospital of the School of Medicine, Xizang Minzu University, China. The key inclusion criteria were based on clinical symptoms, epidemiology, and pathophysiology findings [28]. The clinical features recorded were: age at diagnosis, gender, radiological results, chest sounds, body temperature, heart rate, and oxygen saturation. All HAPE patients eventually exhibited chest infiltrates consistent with pulmonary edema. Controls (n=304) were healthy people selected from the same geographic region as the HAPE cases and recruited from the Hospital of the School of Medicine, Xizang Minzu University, China. The controls did not develop any symptoms or signs of HAPE or any high pressure-related diseases after exposure to high altitude within 7 days. All participants were of Chinese Han population and resided at low altitudes less than 2000 m. No participants used prophylactic medications, and the rate and altitude of ascent were the same among the HAPE cases and controls (altitude of the Tibetan plateau is 4000 to 5000 m). Venous blood samples were obtained from the same hospital to avoid definite selection bias, and the blood samples were taken according to the study protocol approved by the Ethics Committee of Mental Health Center, from the Hospital of School of Medicine, Xizang Minzu University. Informed consent for genetic testing was obtained from each participant before the enrollment of the subjects.

### SNP selection and genotyping

We selected 15 putative functional SNPs in *ADRB2*, *GNB3*, *TH* and *GSTP1* genes. The SNPs were found from recent respiratory publications. The first report was an acute mountain sickness susceptibility study in Nepalese population; from that study, SNPs in *ADRB2* (rs2400707, rs12654778, rs11168070, and rs1042718) were selected [29]. The second report suggested that the *TH* gene may be associated with homovanillic acid and 3-methoxy-4-hydroxyphenylglycol, and the SNPs selected were rs10770140, rs10770141, and rs10840491 [30]. Finally, other studies identified the remaining SNPs associated with lung function [27, 31–33]. The SNPs were identified using dbSNP database (http://www.ncbi.nlm.nih.gov/projects/SNP), and genotyped using Sequenom MassARRAY RS1000 (Sequenom, Inc) following the manufacturer's instructions. Primers were designed for each SNP and are listed in Table 4. Genotyping and data management were performed at the Molecular Research Laboratory of the Biotechnology, Northwest University, China. The overall success rate of all the genotyping assays was over 98%. All samples with ambiguous results were measured repeatedly, as was a random selection of 10% of all samples to ensure laboratory quality control.

**Table 4:** The SNPs primers used for this study

SNP	1st_PCRP	2st_PCRP	UEP_SEQ
rs17778257	ACGTTGGATGCAGTTTTTCAAAGACACCAC	ACGTTGGATGCTTTCCATCTGGCATGTGAC	GTGAAATTAATTTTCAGGGTTTGA
rs2400707	ACGTTGGATGTAAGTCACAGACGCCAGATG	ACGTTGGATGTCCTTTCATCTGCTGGATAG	GTTTGTTAATCTTTCGGGTTG
rs17108803	ACGTTGGATGAAGCACAAAGACATGGTGAC	ACGTTGGATGTGTCTGTGTCGCTCTGGATG	ccctCCAGCGTGTGTTTACTT
rs12654778	ACGTTGGATGGTGTGTCTCAGTGTCTATGG	ACGTTGGATGAGGCACAAATACACCCTGGC	ccctcCACCCTGGCAGACATGCT
rs11168070	ACGTTGGATGACAGAAGAGCCAAAAGCTCC	ACGTTGGATGAGAGGGCTAAAGCTGGAGG	gaTAAAGCTGGAGGTGGTGT
rs1042718	ACGTTGGATGTGAAGAAGTCACAGCAGGTC	ACGTTGGATGTCTTGCCCATTCAGATGCAC	attgaCCATTCAGATGCACTGGTAC
rs3842727	ACGTTGGATGAGCCACGTGACAGTGGGAG	ACGTTGGATGAGCCAGCACAGTTTGTGACC	TGTGACCCCTGCTCCCT
rs2070762	ACGTTGGATGTCCTTCTCACGGATGGTGTC	ACGTTGGATGTCACAGATGAGAAAACCGAC	TGAGAAAACCGACCCCTGG
rs10770140	ACGTTGGATGTAGGAGTGCCATCTGCCCA	ACGTTGGATGCCCTCCTGGGACATTCTGG	ttttGGACATTCTGGACCCCAC
rs10770141	ACGTTGGATGTCTCCAAGGGGAAGGCATCA	ACGTTGGATGACTGCTAGCTCCTGGCTTC	CCTGTGGCCCCTTCTTT
rs10840491	ACGTTGGATGAGAGTGGGCCCTGAGAGATG	ACGTTGGATGAAGCCCTAGACGCTCCCTGA	AGACGCTCCCTGACTTCTC
rs7119275	ACGTTGGATGCACTGGGTGCTGAGAGACA	ACGTTGGATGTGCCACGATATTAATGCCCC	cCCCCTCCCTTGCGCTCC
rs749174	ACGTTGGATGTTCAGACTTCTCAATGGCCC	ACGTTGGATGAGGTCCCGAAGGCCTTGAA	tcttTCCCGAAGGCCTTGAACCCACT
rs4963516	ACGTTGGATGTCTCGCTACCACTTCCTACT	ACGTTGGATGTGCTCTGCAAGTTGAAGCTG	ccccaATTTGCCCCAAGCTAGTCCC
rs5446	ACGTTGGATGTGGGTGGTATAGGGCGTTTG	ACGTTGGATGAGCATGAATAAGAAGAGGGC	ccccAGAAGAGGGCCAGGACCCTAGT

### Statistical analysis

Statistical analyses were performed using SPSS version 17.0 for Windows (SPSS, Chicago, IL) and SNPstats software platform (http://bioinfo.iconcologia.net/SNPstats_web). Analysis of variance (ANOVA) was used to compare differences between genotypes for the various parameters. Chi-square test was used to identify departures from Hardy–Weinberg equilibrium. Analysis for genotype and allele effects was undertaken using the Chi-square test of independence, OR and 95% CI were calculated from the combined results of both populations, adjusting for age and gender. In addition, comparison of population characteristics was performed using an independent sample t-test (using Levene's test for equality of variances) for continuous variables and an additional Chi-square test for categorical variables.
